# In reply to the letter to the editor regarding “The efficacy and safety of tranexamic acid in high tibial osteotomy: a systematic review and meta-analysis”

**DOI:** 10.1186/s13018-021-02628-7

**Published:** 2021-08-16

**Authors:** Jimin Ma, Hanli Lu, Xinxing Chen, Dasai Wang, Qiang Wang

**Affiliations:** 1 Department of Orthopedics, Fuyang Hospital of Anhui Medical University, Fuyang, Anhui China; 2 Department of Orthopedics, Yijishan Hospital of Wannan Medical College, Wuhu, Anhui China

**Keywords:** Blood loss, Hemoglobin decrease, Drain output, High tibial osteotomy, Tranexamic acid, Meta-analysis

Dear Editor,

We appreciate all of the reviewers for their thought-provoking comments about our recent meta-analysis published in the journal [1]. Our answers to these questions raised are as follows:

Firstly, although the heterogeneity of total blood loss is high, we use the random effects model that allows reliable pooled results; this heterogeneity might be attributed to different surgical procedures. At present, there is no conclusion about the data-base to be searched. Many meta-analyses also searched only two or three databases [2–11]. The three databases we searched (PubMed, Embase and Cochrane Library) can find most of the literatures. The article on Ma [12] cannot be included in the above three databases. Also, it was published later than our deadline for retrieval.

It is well known that the prevalence of knee osteoarthritis is much higher in female. Meta-regression analysis for gender as an item does not make much sense. We do not think it is appropriate to conclude that female might benefit more than male on blood management from tranexamic acid (TXA) by dividing them into three subgroups based on gender. From their results, it can only be concluded that no matter what the proportion of female is, TXA can benefit. Moreover, gender difference could affect total blood loss, with greater amount in men compared with women [13–15].

Secondly, we agree with the hypothesis that intraoperative TXA had a short-time effect, but it might benefit patients for a relatively long time. This is consistent with the results of our meta-analysis, which is also in agreement with previous studies [16, 17].

Finally, most of the studies included in this meta-analysis are cohort studies, which cannot be as consistent as randomized controlled trials (RCTs). And we choose random-effect model to make the results tend to be conservative. Moreover, the results were consistent even with the fixed-effect model (Figs. 1 and 2). The Stata 15.0 software (StataCorp, College Station, TX, USA) was performed to evaluate the publication bias. The results of the funnel plot “Egger test” (*P* = 0.247) indicated a low risk of publication bias. However, as this meta-analysis did not include enough studies, the reliability of these assessments was not very strong. Strictly speaking, publication bias in this study is not necessary.

**Fig. 1:**
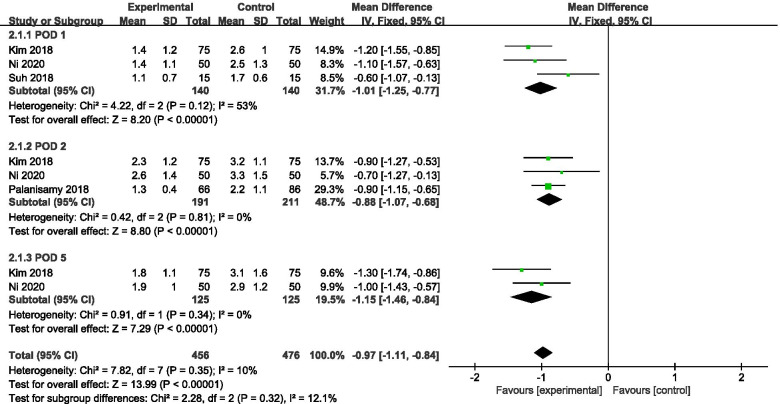
Forest plot showing the hemoglobin decrease of patients undergoing HTO between TXA and control groups

**Fig. 2:**
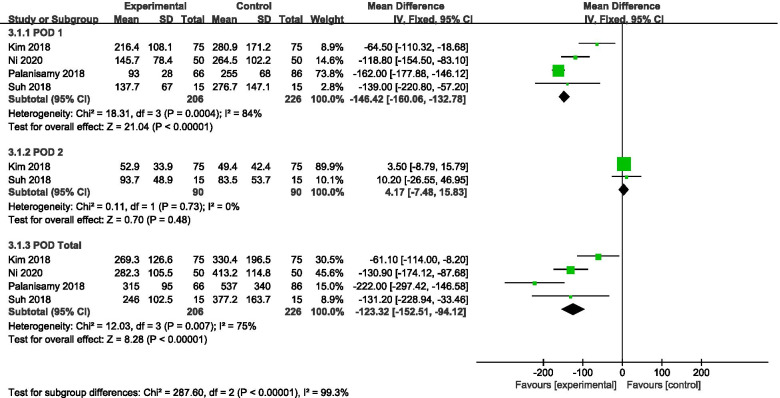
Forest plot showing the drain output of patients undergoing HTO between TXA and control groups

## Data Availability

All data generated or analyzed during this study are included in this published article.

## Abbreviations

TXA: Tranexamic acid
RCTs: Randomized controlled trials
